# Vitamin D and cardiovascular autonomic neuropathy in type 2 diabetes mellitus according to diabetic kidney disease stage

**DOI:** 10.3389/fendo.2026.1810159

**Published:** 2026-04-15

**Authors:** João Felício, Ester Chambouleyron, Valéria Nascimento, Isabel Fernandes, Lícia Ruivo, Maria Vitória Hupp, Lilian Silva, Natércia de Queiroz, Franciane de Melo, Ana Carolina de Souza, Márcia dos Santos, Lorena de Moraes, Mayana Barros, Danielle Pinheiro, Maria Eduarda do Carmo, Beatriz Lobo, Pedro Piani, Ana Regina Motta, Melissa dos Reis, Valéria Suênya Leal, Priscila de Figueiredo, Karem Felício

**Affiliations:** Endocrinology Division, University Hospital João de Barros Barreto, Federal University of Pará, Belem, Brazil

**Keywords:** cardiovascular autonomic neuropathy, diabetic neuropathy, diabetic kidney disease, Type 2 diabetes melitus, vitamin D

## Abstract

**Background/objectives:**

Cardiovascular autonomic neuropathy (CAN) may be associated with other diabetes mellitus-related complications. In addition, lower vitamin D (VD) levels have been associated with diabetic kidney disease (DKD) and diabetic neuropathy. We evaluated the relationship between serum VD and CAN in patients with type 2 Diabetes Mellitus (T2DM) in early and advanced stages of DKD.

**Methods:**

Seventy-six T2DM patients, 28 in early DKD stage (urine albumin to creatinine ratio (UACR)): 30 to 299 mg/g - group 1), and 48 in advanced DKD stage (UACR ≥300 mg/g - group 2), participated.

**Results:**

In group 1, prevalence of CAN was 46% versus 75% in group 2 (p=0.01). 25(OH)D was lower in group 2 (26.3 ± 9.8 vs 30.0 ± 8.0; p<0.05) and, in this group, those with CAN vs without CAN showed lower 25(OH)D (27.8 ± 8.3 vs 32 ± 6.3; p<0.05). Only in group 2, patients with VD deficiency (<no><20</no> ng/ml) vs normal, showed worse CAN parameters, particularly VLF (65.5 (46-104) vs 309 (106.5-682.5), p<0.01), SDNN (10.5 (8-17.5) vs 28.5 (13-48), p<0.05) and Valsalva Maneuver (1.12 ± 0.04 vs 1.30 ± 0.21, p<0.05). We have found a correlation between VD concentration and CAN prevalence (r = -0.3, p<0.05). Logistic regression showed that VD concentration <no><20</no> ng/ml increased 24 times the chance of abnormal VLF (R²: 0.38; OR: 24.1; 95% (CI: 2.6–222); p<0.01).

**Conclusions:**

To our knowledge, this is the first study to demonstrate an association between lower VD and CAN in T2DM and advanced DKD.

## 1 Introduction

Diabetic neuropathy is the most prevalent and underdiagnosed chronic complication in individuals with type 2 diabetes mellitus (T2DM) (1, 2). Among these, cardiovascular autonomic neuropathy (CAN) is of particular clinical relevance, due to alterations in heart rate variability (HRV) and its association with higher morbidity and mortality in T2DM individuals, particularly when associated with diabetic kidney disease (DKD) (3–6). In individuals with T2DM, the prevalence of cardiovascular autonomic neuropathy (CAN) ranges from 12% to 73% (7), while vitamin D deficiency affects approximately 64.2% of this group (8). Both complications have been associated with reduced vitamin D (VD) concentrations in cross-sectional studies (9–14). There is also evidence suggesting that DKD and CAN may improve after VD supplementation (15–19).

Vitamin D deficiency has been associated with increased inflammation, oxidative stress, and reduced neurotrophin availability, mechanisms that may contribute to the development of CAN (20–22). Considering DKD, a progressive renal dysfunction characterized by worsening albuminuria and impaired VD activation, this condition may coexist with and intensify these pathophysiological pathways, in parallel with greater autonomic dysfunction across more advanced stages of kidney disease (23, 24).

Recently, our research group demonstrated improvements in HRV (in both frequency and time domain) after vitamin D supplementation in patients with type 1 diabetes mellitus (T1DM) (18). We have also found an improvement in morning systolic blood pressure and CAN parameters after vitamin D supplementation (19). Furthermore, loss of nocturnal systolic blood pressure (NSBP) dipping is a condition that increases cardiovascular risk and is associated with both CAN and DKD (19, 25). Although these findings provide an important conceptual basis, differences in pathophysiology require specific investigation in T2DM (26, 27). Finally, to our knowledge, no study has evaluated a possible association between lower VD concentrations and CAN in all DKD stages in the same T2DM population.

## 2 Materials and methods

### 2.1 Study design and patients

This was a cross-sectional study designed to evaluate potential associations between serum VD concentrations and CAN in patients with T2DM at early and advanced stages of DKD. Seventy-six patients with T2DM, 28 in early DKD stage (UACR: 30 a 299 mg/g - group 1) and 48 in advanced DKD stage (UACR ≥ 300 mg/g - group 2) of DKD, were recruited from the Endocrinology Department of the João de Barros Barreto University Hospital (HUJBB), Federal University of Pará (UFPA), between 2023 and 2025. Informed Consent Form (ICF) was obtained from all participants. The study was conducted in accordance with the Declaration of Helsinki and the Nuremberg Code and was approved by the Ethics Committee of the João de Barros Barreto University Hospital (reference number 88974918.6.0000.0017). The gold standard established by the Toronto Consensus, based on cardiovascular autonomic reflex tests (CARTs), was used to assess the presence of CAN in these patients (3).

Inclusion criteria were: (a) provision of ICF prior to any study procedure; (b) age >30 years and diagnosis of T2DM under regular follow-up with an endocrinologist; (c) treatment with stable doses of oral hypoglycemic agents or insulin for at least three months; (d) presence of UACR ≥ 30 mg/g; (e) estimated glomerular filtration rate (GFR) between 25 and 90 mL/min/1.73 m²; (f) stable doses of all medications for at least four weeks prior to the screening visit; (g) ability and willingness to undergo all study procedures; (h) ability and willingness to attend scheduled visits and complete all required procedures.

Exclusion criteria were: (a) T1DM or other diabetes types; (b) history of bone metabolism disorders; (c) history of liver disease; (d) use of vitamin D or calcium supplements within the last three months before screening; (e) uncontrolled hypothyroidism or hyperthyroidism; (f) pregnancy, intention to become pregnant, or breastfeeding; (g) comorbidities likely to affect life expectancy, according to the investigator; (h) consumption of alcohol or recreational drugs that could compromise patient safety or study procedures; (i) clinically significant arrhythmias that could interfere with CARTs; (j) indication for dialysis initiation or current dialysis treatment.

All patients were followed at the Endocrinology Ambulatory and received orientation about diet and exercise according to American Diabetes Association (ADA) guidelines (28).

### 2.2 Data collection

After eligibility confirming, patients who met all inclusion criteria underwent the study procedures. All participants had a well-established clinical and laboratory diagnosis of T2DM (27). Data collection occurred during scheduled visits and included medical records review (pre-existing clinical conditions, demographics, insulin use, and other current medications), physical examination, laboratory tests, and CAN assessment. Nephropathy and neuropathy were evaluated according to ADA guidelines (1, 29). Serum 25(OH)D concentrations were determined using the DiaSorin LIAISON 25-OH-Vitamin D TOTAL chemiluminescence immunoassay (DiaSorin, Stillwater, MN, USA) (30). This method is part of the techniques evaluated by the Vitamin D External Quality Assessment Scheme (DEQAS), the largest specialized external quality control (proficiency testing) program for the measurement of vitamin D metabolites, including 25(OH)D and 1,25(OH)_2_D.

Hemoglobin A1c (HbA1c) was measured using high-performance liquid chromatography (HPLC) (31). Fasting plasma glucose, phosphorus, total calcium, urea, albumin, total cholesterol and fractions (low-density lipoprotein (LDL) and high-density lipoprotein (HDL) and triglycerides) were analyzed using automated colorimetric methods. Creatinine was measured using kinetic/automated methods, and albuminuria by immunoturbidimetry (32). Glomerular filtration rate (GFR) was estimated using the CKD-EPI equation (33), which was refitted without race variables (34, 35). UACR was assessed in three separate spot urine samples.

### 2.3 CAN assessment

In order to evaluate CAN, CARTs and HRV analysis were performed with the following pre-recording considerations: tests were performed in the morning after confirming capillary glucose levels between 70 and 250 mg/dL; participants were instructed to abstain from alcohol and caffeine and to refrain from smoking for at least eight hours prior to testing; they were also asked to suspend antihypertensive, anxiolytic, antidepressant, decongestant medications and avoid vigorous physical activity for 24 hours; tests were rescheduled if the patient had fever (>37.8 °C) within the previous two days, significant emotional stress the day before, or hypoglycemia within eight hours of testing.

#### 2.3.1 CARTs evaluation

CARTs, considered the gold standard according to the Toronto Consensus, included Deep Breathing, Valsalva Maneuver, and Orthostatic Test (30:15 ratio and Orthostatic Hypotension) (3). Valsalva Maneuver was performed with the patient supine, maintaining an expiratory pressure of 40 mmHg for 15 seconds, with heart rate response evaluated by ECG. The orthostatic (30:15) test assessed the ratio between maximal tachycardia and subsequent bradycardia after standing. The deep breathing test evaluated the E:I ratio during controlled inspiration and expiration. Orthostatic hypotension was defined as a ≥20 mmHg systolic and/or ≥10 mmHg diastolic blood pressure reduction within 3 minutes of standing.

#### 2.3.2 HRV evaluation

Heart rate variability (HRV) time-domain parameters were obtained with patients in the supine position. HRV was assessed using a computerized system (VNS-MICRO) (36). For spectral analysis in the three frequency bands—VLF, LF, and HF—the patient remained in the supine position at 30 degrees with spontaneous breathing. A 300-second ECG recording was obtained. The signal was processed by mathematical algorithms generating amplitude-versus-frequency plots. HRV spectral components included: Very Low Frequency (VLF: 0.01–0.04 Hz), related to vasomotor tone, thermoregulation, and sweating (sympathetic control); Low Frequency (LF: 0.04–0.15 Hz), associated with baroreflex activity (sympathetic with vagal modulation); High Frequency (HF: 0.15–0.5 Hz), associated with parasympathetic (vagal) control.

Time-domain parameters included: RRmin, RRmax, RRNN (mean NN interval), and SDNN (standard deviation of all NN intervals) (37, 38). Frequency domain parameters were composed of: VLF, HF, LF and Total Power (TP), a set of three combined spectral bands and LF/HF ratio (which reflects the balance between sympathetic and parasympathetic activity). Although it is not a diagnostic criterion, it provides additional information on sympathetic and parasympathetic performance. In each test the relation between the largest and smallest RR interval is assessed and then, a coefficient was obtained.

Regarding HRV indices and time-domain parameters, no analyses including large healthy populations have established definitive reference values. Therefore, the values proposed by Angelink et al. (2001) (36), which are based on studies with relatively small sample sizes, were adopted. Consequently, HRV indices and time-domain parameter values should be considered approximate and should not be used as the sole basis for clinical decision-making. The presence or absence of CAN was defined according to the Toronto Consensus (3).

### 2.4 DKD Classification

All patients had their urine albumin-to-creatinine ratio measured three times, each at different time points. Three categories were considered: normal UACR (<30 mg/g), early stage (UACR 30–299 mg/g), and advanced stage (UACR ≥300 mg/g) of DKD (39). Each patient was classified according to the category observed in at least two of the three assessments, and only those with early stage (group 1) or advanced stage (group 2) of DKD were included. For clarity and consistency throughout the manuscript, patients with increased and severely increased albuminuria were classified as having early and advanced DKD stages.

### 2.5 Vitamin D Assessment

For the analysis of serum vitamin D concentration, the criteria established by the Institute of Medicine (IOM) guideline was used. According to these guidelines, 25(OH)D concentrations were classified as deficiency (<20 ng/mL) or normal (≥20 ng/mL) (40).

### 2.6 Statistical analysis

Statistical analyses were performed according to the distribution of the variables (normality determined using the Shapiro–Wilk test) and statistical significance was considered when the null hypothesis was rejected at p < 0.05 in all tests. Student’s t-test and one-way ANOVA were used for normally distributed variables when comparing two or more groups, respectively. For non-normally distributed variables, the Mann–Whitney test was used for independent samples, and the Kruskal–Wallis test was applied when comparing more than two groups. Chi-square or Fisher’s exact tests were applied for categorical variables, and data are reported as absolute values and frequencies (%). For normally distributed variables, data are reported as mean ± standard deviation, and for non-normally distributed variables, data are reported as median (25th–75th percentile). Spearman or Pearson correlation tests were performed. A logistic regression analysis was performed using VLF test normal or abnormal as dependent variable and VD concentration (<20 ng/mL vs ≥20 ng/mL) as the independent variable. In addition, a multivariable logistic regression analysis was performed using VLF test as dependent variable and VD concentration as the independent variable, to adjust for potential confounding variables (age, T2DM duration, HbA1c, GFR, lipid profile, blood pressure and use of angiotensin-converting enzyme inhibitors (ACEi) and angiotensin II receptor blockers (ARB), SGLT2 (Sodium-Glucose Cotransporter-2) inhibitors, beta-blockers and glucagon-like peptide-1 (GLP-1) agonists). A statistical power > 0.8 for comparisons between variables of interest was considered acceptable. The sample size was estimated first to find a difference between VD concentration in different stages of DKD using t-test and considering an expected difference in means = 4 ng/mL, an expected standard deviation = 8 ng/mL and a desired power = 0.8, with p < 0.05. A minimum sample size of 64 individuals was estimated. In addition, we estimated a sample size of 70 individuals to achive a desired correlation between CAN prevalence and VD concentration, considering a desired power = 0.8, p < 0.05 and a correlation coefficient at least of 0.3.

Collected data were organized and analyzed using SigmaStat 3.5^®^ (Jandel Scientific Corporation, Chicago, Illinois) and the Statistical Package for the Social Sciences (SPSS 22^®^).

## 3 Results

The demographic and clinical characteristics of the 76 recruited patients are presented in **Table 1**. Group 2, comprising individuals with advanced-stage DKD, showed a higher prevalence of chronic diabetes-related complications, particularly a prior history of diabetic neuropathy and CAN, as well as a longer duration of T2DM—findings that are expected in patients with more severe renal impairment (**Table 1**).

**Table 1:** Clinical parameters of patients with early-stage (Group 1) and advanced-stage (Group 2) of DKD.

Clinical features	Group 1	Group 2	*p*
	n = 28	n = 48	
Age (years)	63.8 ± 8.3	66.0 ± 6.6	0.19
Gender (M/F)	15/13	21/27	0.48
BMI (kg/m²)	30.8 ± 4.9	29.6 ± 4.1	0.46
Time since diagnosis of T2DM (years)	9.14 ± 6.2	16.8 ± 8.4	<0.001
Dyslipidemia (yes%)	23 (88.2)	44 (91.6)	0.14
Previous cardiovascular event (yes%)	4 (14.8)	5 (10.4)	0.71
History of previous neuropathy (yes%)	16 (57.1)	44 (91.6)	<0.001
History of retinopathy (yes%)	4 (14.3)	8 (16.7)	0.78
History of smoking (yes%)	11 (39.3)	16 (33.3)	0.69
History of alcoholism (yes%)	11 (39.3)	24 (50.0)	0.17
Autonomic neuropathy (yes total%)	13 (46.4)	36 (75.0)	0.01
Use of ACEi/ARBs (yes total%)	27 (96.4)	48 (100.0)	0.37
Use of beta-blockers (yes total%)	6 (21.4)	17 (35.4)	0.30

M, Male; F, Female; BMI, Body-mass index; T2DM, Type 2 diabetes mellitus; ACEi, Angiotensin-Converting Enzyme Inhibitors; ARB, Angiotensin II Receptor Blockers.

**Table 2** shows laboratory parameters of both groups: lower 25(OH)D concentrations were observed in group 2 compared with group 1. In addition, patients in the advanced DKD group exhibited poorer glycemic parameters, as reflected by higher fasting plasma glucose concentrations (mg/dL), although HbA1c did not differ significantly between groups. Furthermore, as expected regarding renal function, participants in group 2 had higher serum creatinine levels, lower GFR and higher albuminuria levels (**Table 2**).

**Table 2:** Laboratory parameters of patients with early-stage (group 1) and advanced-stage (group 2) of DKD.

Laboratory features	Group 1	Group 2	*p*
	n = 28	n = 48	
25(OH)D (ng/mL)	30.0 ± 8.0	26.3 ± 9.8	0.03
Glucose (mg/dL)	130.7 ± 40.2	161.6 ± 61.2	0.02
HbA1c (%)	7.9 ± 0.9	8.2 ± 1.3	0.17
Creatinine (mg/dL)	0.84 ± 0.24	1.4 ± 0.4	<0.001
GFR (CKD-EPI) (mL/min/1,73m²)	88.3 ± 17.4	51.2 ± 14.4	<0.001
Total cholesterol (mg/dL)	160.0 ± 46.2	187.2 ± 48.7	0.02
HDL (mg/dL)	40.6 ± 12.5	40.4 ± 10.5	0.93
LDL (mg/dL)	86.3 ± 35.3	100.8 ± 43.3	0.15
Triglycerides (mg/dL)	171.0 ± 84.9	233.6 ± 153.0	0.05
Mean albuminuria (mg/g)	105.0 ± 67.7	614.0 ± 585.0	<0.001
Mean albuminuria (log10)	1.9 ± 0.3	2.6 ± 0.5	<0.001

25(OH)D, 25-hydroxyvitamin D; HbA1c, Glycated hemoglobin; GFR, Glomerular Filtration Rate; CKD-EPI, Chronic Kidney Disease Epidemiology Collaboration; HDL, High-density lipoprotein; LDL, Low-density lipoprotein.

Laboratory parameters of patients with advanced-stage DKD stratified according to the presence or absence of CAN are in **Table 3**. In this analysis, no significant differences were observed between groups for glycemic control, renal function, albuminuria or lipid profile. However, VD concentrations were significantly lower in the group with CAN when compared with those without CAN.

**Table 3:** Laboratory parameters of group 2 patients with and without CAN.

Laboratory features	Without CAN	CAN	*p*
	n = 12	n = 36	
25 (OH)D (ng/mL)	32 ± 6.3	27.8 ± 8.3	0.04
Glucose (mg/dL)	147.7 ± 33.9	166.3 ± 67.7	0.22
HbA1c (%)	8.3 ± 1.4	8.2 ± 1.2	0.84
Creatinine (mg/dL)	1.5 ± 0.6	1.4 ± 0.3	0.50
Clearance (CKD-EPI) mL/min/1,73m²)	47.8 ± 16.5	52.4 ± 12.7	0.33
Total cholesterol (mg/dL)	182.4 ± 46.0	188.8 ± 50.7	0.70
HDL (mg/dL)	41 (34–43)	37 (31 – 45)	0.60
LDL (mg/dL)	100.8 ± 52.2	100.8 ± 41.4	1.00
Triglycerides (mg/dL)	212 ± 117.4	239.7 ± 166.5	0.60
Mean albuminuria (mg/g)	652.1 (170.8 - 1390.2)	455 (179.4-733.9)	0.23
Mean albuminura (log10)	2.8 (2.2 - 3.1)	2.7 (2.3 - 2.9)	0.23

25(OH)D, 25-hydroxyvitamin D; HbA1c, Glycated hemoglobin; CKD-EPI, Chronic Kidney Disease Epidemiology Collaboration; HDL, High-density lipoprotein; LDL, Low-density lipoprotein.

The differences in CAN parameters between groups 1 and 2 are shown in **Table 4**. Worse values were observed in group 2, representing advanced-stage DKD, in both frequency- and time-domain parameters (SDNN), as well as across all CARTs (Deep breathing, Orthostatisc (30:15) test and Valsalva Maneuver). The statistical power of these comparisons was acceptable (power > 0.8).

**Table 4:** CAN parameters in patients with early stage (group 1) and advanced stage (group 2) of DKD.

Parameters	Group 1	Group 2	*p*
	n = 28	n = 48	
Frequency domain parameters			
TP (ms2)	1114 (472 – 2905)	548 (131 – 1552)	0.03
VLF (ms2)	350 (203 – 565)	233 (79.8 - 612)	0.15
LF (ms2)	429 (105 – 1020)	71 (24.5 - 326)	<0.01
HF (ms2)	306 (86.8 - 1381)	110 (17.5 - 534)	0.03
LF/HF (ms2)	1.0 (0.6 - 1.9)	0.7 (0.4 - 1.7)	0.22
Time domain parameters			
RRmin (ms)	660 (291- 763)	698 (587 - 810)	0.09
RRmax (ms)	912 (820 - 1191)	955 (779 - 1135)	0.96
RRNN (ms)	804 (751 - 895)	861 (732 - 977)	0.40
SDNN (ms)	34 (21 - 53.8)	25.5 (11.0 - 46.3)	0.05
Autonomic cardiac reactivity tests			
Deep breathing	1.18 ± 0.17	1.10 ± 0.10	0.02
Orthostatic (30:15) test	1.13 ± 0.13	1.07 ± 0.08	0.01
Valsalva Maneuver	1.56 ± 0.46	1.27 ± 0.20	<0.01

TP, Total power; VLF, Very low frequency; LF, Low frequency; HF, High frequency; RRmin, Minimum RR interval; RRmax, Maximum RR interval; RRNN, Mean length of all normal-to-normal RR intervals; SDNN, Standard deviation of all normal-to-normal RR intervals.

Comparisons were performed for CAN parameters according to VD concentration, with serum categories defined according to the Institute of Medicine guidelines (**Table 5**). Exclusively in group 2, frequency domain parameters (TP, VLF and LF), time domain parameters (SDNN) and autonomic cardiac reactivity tests (Deep breathing, Orthostatic (30:15) test and Valsalva Maneuver) showed significant differences between the deficiency VD and normal VD groups. No significant differences according to vitamin D concentration were observed in group 1. The power of those comparisons was acceptable (power > 0.8).

**Table 5:** Association between CAN parameters and vitamin D concentration according to the Institute of Medicine in group 1 and group 2.

Parameters	Group 1 (n = 28)		*p*	Group 2 (n = 48)		*p*
	Deficiency VD (<20 ng/mL)	Normal VD (≥20 ng/mL)		Deficiency VD *p* (<20 ng/mL)	Normal VD (≥20 ng/mL)		
	n = 4	n = 24		n = 8	n = 40		
Frequency domain parameters						
TP (ms2)	743 (154.5-3835)	1365 (493-3165)	0.34	107.5 (74-291.5)	884.5(211-2276.5)	0.01
VLF (ms2)	264 (93-420)	350 (205-646)	0.28	65.5 (46-104)	309 (106.5-682.5)	<0.01
LF (ms2)	326 (40-784)	473 (115-1099)	0.31	20.5 (4.5-48)	102 (32.5-370)	0.02
HF (ms2)	153 (22-2630)	400 (93-1412)	0.56	16 (9-159.5)	175 (25-731.5)	0.06
LF/HF (ms2)	2 (1-3)	0.90 (1-2)	0.53	0.81 (0.32-1.2)	0.69 (0.36-2.1)	0.71
Time domain parameters						
RRmin (ms)	641 (370- 940)	661 (290-770)	0.61	707.5 (608.5-913.5)	698 (576-796)	0.65
RRmax (ms)	1021 (702-1586)	912 (822-1170)	0.82	947.5 (697-1028.5)	957 (796.5-1162.5)	0.31
RRNN (ms)	782 (675-1070)	804 (755-902)	0.72	893.5 (662-980.5)	844.5 (745.5-978)	0.54
SDNN (ms)	26 (10-70)	35 (21-55)	0.38	10.5 (8-17.5)	28.5 (13-48)	0.02
Autonomic cardiac reactivity tests						
Deep breathing	1.11 ± 0.13	1.19 ± 0.17	0.36	1.05 ± 0.04	1.12 ± 0.11	0.04
Orthostatic (30:15) test	1.14 ± 0.11	1.13 ± 0.13	0.87	1.03 ± 0.02	1.08 ± 0.08	0.01
Valsalva Maneuver	1.38 ± 0.25	1.59 ± 0.49	0.45	1.12 ± 0.04	1.30 ± 0.21	<0.05

VD, Vitamin D; TP, Total power; VLF, Very low frequency; LF, Low frequency; HF, High frequency; RRmin, Minimum RR interval; RRmax, Maximum RR interval; RRNN, Mean length of all normal-to-normal RR intervals; SDNN, Standard deviation of all normal-to-normal RR intervals.

We also found a correlation between serum VD concentration and the prevalence of CAN (r = −0.3, p < 0.05), indicating that lower vitamin D concentrations were associated with a higher likelihood of CAN. Additionally, higher VD concentrations were also associated with better CAN parameters (VLF: r = 0.3, p < 0.05; Orthostatic (30:15) test: r = 0.3, p < 0.05; Valsalva maneuver: r = 0.3, p < 0.05), suggesting that higher vitamin D concentrations were associated with better autonomic performance across these tests.

To further explore these associations, logistic regression analyses were performed using VLF test normal or abnormal as dependent variable and VD concentration (≥ 20 ng/mL and < 20 ng/mL) as the independent variable. It showed that VD concentration < 20 ng/mL increased notably the probability to have an abnormal VLF test (r-square: 0.38; OR: 24.1 (95% CI: 2.61 – 222.63); p < 0.01).

Additionally, multivariable logistic regression analysis was performed, using VLF test as dependent variable and VD concentration as the independent variable. VD concentration remained an independent predictor of abnormal VLF, leading to a worsening of the CAN, independent of the following parameters: age, T2DM duration, HbA1c, GFR, lipid profile, blood pressure and use of ACEi/ARBs, SGLT2 inhibitors, beta-blockers and GLP-1 receptor agonists (r-square: 0.3; p < 0.05).

## 4 Discussion

Our data demonstrated an association between reduced vitamin D concentration and the prevalence and severity of CAN in patients with T2DM and advanced DKD. Those with lower VD concentrations presented impairment in HRV and CART parameters, and this association was not observed in the group with early-stage DKD. In addition, lower VD concentration was correlated with a higher prevalence of CAN and with the worsening of several CAN parameters. Finally, logistic regression analyses indicated that VD concentrations < 20 ng/ml were associated with abnormality in one of the major frequency domain parameters, increasing 24 times the probability of VLF test. In this context, reduced VLF power has been consistently associated with arrhythmic death (41). Previous studies have also demonstrated that lower serum VD levels may be associated with an increased incidence of cardiovascular events and a higher risk of arrhythmic death (42, 43). VLF is believed to reflect slower regulatory mechanisms, including renin–angiotensin system activity, thermoregulation, endothelial function (44, 45), and inflammatory modulation (46, 47). Given that VD deficiency has been linked to renin–angiotensin system activation and pro-inflammatory states, these mechanisms provide biologically plausible pathways connecting lower vitamin D concentrations to impaired VLF modulation (48–50).

Some studies have reported associations between vitamin D with diabetic neuropathies (51–53). Sari et al. found an improvement in painful diabetic neuropathy after VD supplementation in 57 patients with T2DM (51). Basit et al. showed similar results after a single high dose intramuscular VD administration in 143 patients with painful diabetic neuropathy (52). Shehab et al. also described the same findings when short-term oral VD supplementation was used (53). In two systematic reviews with meta-analysis, Gilbody et al. and Yammine et al., including a total of 320 and 364 patients with diabetes, showed that short-term vitamin D supplementation resulted in an improvement in pain scores, however, no significant changes in nerve conduction were reported (54, 55). All these studies were in diabetic patients with peripheral neuropathy and therefore, it is not possible to extrapolate these findings to patients with CAN.

Studies investigating the association between VD and CAN are scarce (56–58). Hansen et al., evaluating the association between serum vitamin D concentration, reflex tests and HRV indices in 113 patients with DM, demonstrated through linear regression that an increase in vitamin D from 10 ng/mL to 20 ng/mL led to improvements in heart rate variability and reflex test performance (deep-breathing response and the 30/15 orthostatic test) (56). Similar findings were described for Jung et al. and Chen et al. studying 163 and 191 patients with T2DM (57, 58). All three of these studies were cross-sectional and, recently, our group performed one longitudinal study in patients with T1DM and found an improvement in CAN parameters after high dose VD supplementation (18, 19). Nevertheless, none of these studies evaluated specifically patients with DM and DKD, which is a condition associated with lower VD concentration and CAN (5, 6, 12, 13, 15, 17, 23, 24, 59–63).

Some studies evaluated the relationship between DKD and VD (12, 13, 15, 17, 59–62). In a cross-sectional population-based study with 1,576 individuals with diabetes, we showed that lower vitamin D concentrations were associated with albuminuria increase with a higher risk of progression to DKD (12). Similarly, Zomorodian et al. revealed that the prevalence of macroalbuminuria was higher in patients with T2DM and deficient VD (59). Shahwan et al. found a greater tendency toward deficient vitamin D concentration among diabetic patients with an GFR < 60 mL/min, a urine albumin-to-creatinine ratio > 30, or serum creatinine > 1.8 mg/dL (13). In addition, De Boer et al. described an association between lower VD concentration and a higher risk of microalbuminuria in T1DM patients (60). Therefore, some cross sectional and cohort studies are consistent in describing an association between lower VD concentration and DKD. Longitudinal studies evaluating VD supplementation effects on DKD were summarized in two meta-analyses: Xuan et al. and Gupta et al., evaluating 10 and 9 studies with a total of 651 and 734 patients, described a possible benefit of VD supplementation on DKD (61, 62). Nevertheless, it needs to be confirmed by more randomized clinical trials. Finally, as well established, the association between CAN and DKD (5, 6, 23, 24, 63), with most of the studies showing that CAN is associated with an increased risk and progression of kidney damage (5, 23, 63). However, despite previously describing an association between CAN and DKD and some evidences showing that lower VD concentration could play a role in those two diabetic complications pathophysiological mechanisms, no studies have examined the association between VD status and CAN across different stages of DKD in patients with type 2 diabetes in the same population.

Gembillo et al. linked vitamin D deficiency and increased expression of pro-inflammatory cytokines (48). Additionally, VD deficiency has been associated with the pathophysiology of DKD, and, as renal mass declines, there is a concomitant reduction in the availability of 1-α-hydroxylase — the enzyme responsible in renal tissue for converting 25-hydroxyvitamin D into its active form, 1,25-dihydroxyvitamin D — thereby leading to decreased vitamin D availability, which may be associated with increased activation of the renin–angiotensin–aldosterone system (49, 50). In parallel, studies have shown that vitamin D deficiency increases inflammatory markers such as C-reactive protein, interleukins, and toll-like receptors 2 and 4, thereby contributing to the development of CAN. In addition, reductions in neurotrophins — growth factors essential for protecting nerves against oxidative stress — have been linked to lower serum vitamin D concentration, resulting in decreased neurotrophin availability and rendering nerves more susceptible to oxidative damage and inflammation (20–22, 64). It may suggest that VD could be involved in both diabetic microvascular complications, through similar or different mechanisms. Our data showed that, in the group with advanced DKD stage, patients with CAN had lower vitamin D concentrations compared to those without CAN, suggesting that the presence of advanced DKD may modify or exacerbate the relationship between CAN and vitamin D. Nevertheless, it needs to be confirmed by larger studies.

The main limitation of our study is the relatively small sample size, which needs to be increased. The main strength was the homogeneity of our population, separated into different stages of DKD, excluding dialysis and pre-dialysis patients, and also the standardization of CAN diagnosis. Given its cross-sectional design, it is not possible to establish a causal relationship between vitamin D levels and CAN in patients with diabetic kidney disease, and the possibility of reverse causality cannot be excluded. Dietary intake, physical activity, and sunlight exposure may represent potential sources of confounding. To minimize this effect, diet and physical activity were standardized for all participants according to ADA guidelines (28), nevertheless, these factors may still contribute to residual confounding. In our region, sunlight exposure is relatively intense and consistent throughout the year, with no well-defined seasons, which likely has limited study impact. As we are aware, this is the first study to demonstrate an association between lower vitamin D levels and CAN in T2DM and advanced DKD stage. It was not observed in the group in the early DKD stage.

These findings may have important clinical implications. The association between vitamin D deficiency and worse CAN parameters, particularly in patients with DKD, suggests a potential role of vitamin D in this high-risk population. Accordingly, our findings suggest that reduced VD concentrations may be useful for cardiovascular risk stratification, identification of DKD subgroups more vulnerable to autonomic nervous system impairment, and for enabling more targeted monitoring, as well as for generating hypotheses for future interventional studies in this population.

## 5 Conclusions

To our knowledge, this is the first study to demonstrate an association between lower vitamin D levels and CAN in T2DM and advanced DKD stage. It was not observed when patients were in the initial stage of renal disease. Finally, as we used IOM guidelines, the cutoff value for VD concentration that showed worse CAN parameters was 20 ng/ml. As our study was cross-sectional, we can´t establish causal implications and we need a prospective trial to establish if the maintenance of VD concentration must be above this value could bring benefit to these patients.

## Data Availability

The raw data supporting the conclusions of this article will be made available by the authors, without undue reservation.
